# PIMP Your Stride: Preferred Running Form to Guide Individualized Injury Rehabilitation

**DOI:** 10.3389/fresc.2022.880483

**Published:** 2022-05-31

**Authors:** Cyrille Gindre, Bastiaan Breine, Aurélien Patoz, Kim Hébert-Losier, Adrien Thouvenot, Laurent Mourot, Thibault Lussiana

**Affiliations:** ^1^Research and Development Department, Volodalen Swiss Sportlab, Aigle, Switzerland; ^2^Department of Movement and Sports Sciences, Ghent University, Ghent, Belgium; ^3^Institute of Sport Sciences, University of Lausanne, Lausanne, Switzerland; ^4^Department of Sports Science, National Sports Institute of Malaysia, Kuala Lumpur, Malaysia; ^5^Faculty of Health, Sport and Human Performance, University of Waikato, Adams Centre for High Performance, Tauranga, New Zealand; ^6^Research Unit EA3920 Prognostic Markers and Regulatory Factors of Cardiovascular Diseases and Exercise Performance, Health, Innovation Platform, University of Bourgogne Franche-Comté, Besançon, France; ^7^Division for Physical Education, Tomsk Polytechnic University, Tomsk, Russia

**Keywords:** rehabilitation, exercise, running, clinical evaluation, biomechanics

## Abstract

Despite the wealth of research on injury prevention and biomechanical risk factors for running related injuries, their incidence remains high. It was suggested that injury prevention and reconditioning strategies should consider spontaneous running forms in a more holistic view and not only the injury location or specific biomechanical patterns. Therefore, we propose an approach using the preferred running form assessed through the Volodalen^®^ method to guide injury prevention, rehabilitation, and retraining exercise prescription. This approach follows three steps encapsulated by the PIMP acronym. The first step (P) refers to the preferred running form assessment. The second step (I) is the identification of inefficiency in the vertical load management. The third step (MP) refers to the movement plan individualization. The answers to these three questions are guidelines to create individualized exercise pathways based on our clinical experience, biomechanical data, strength conditioning knowledge, and empirical findings in uninjured and injured runners. Nevertheless, we acknowledge that further scientific justifications with appropriate clinical trials and mechanistic research are required to substantiate the approach.

## Introduction

Despite the wealth of research on injury prevention and biomechanical risk factors for running related injuries (RRI), their incidence remains high (1). Inconsistent associations between biomechanical factors and RRI have been observed, both in science and practice (2). As a result, injury prevention and strengthening, reconditioning, or rehabilitation programs in clinical management of runners can be challenging. Recently, Jauhiainen et al. (3) concluded that injury prevention and reconditioning strategies should consider spontaneous running forms in a more holistic view and not only the injury location or specific biomechanical patterns. Other authors suggested that the higher prevalence of soft tissue injuries and lacerations observed in cerebral palsy athletes compared to other disabled athletes could be explained by their moving and walking patterns (4). Herein, we suggest an approach using the preferred running form assessed through the Volodalen^®^ method (5) to guide injury prevention, rehabilitation, and retraining exercise prescription. The approach is based on biomechanical concepts from the scientific literature, as well as our clinical experiences, and evaluates potential discrepancies between spontaneously chosen running forms, biomechanical abnormalities, and the natural tendency for biological systems to self-optimize (6). In using this approach, clinicians need to answer three questions, following three steps, encapsulated by the **PIMP** acronym. The first step is **P**, which stands for **P**referred running form assessment, with the question “Where is the runner on the terrestrial-aerial running form continuum?”. The second step is **I**, which stands for identification of **I**nefficiency in the vertical load management, with the question “Is the running stride too soft, too hard, or deems appropriate?”. The third step is **MP**, which stands for **M**ovement **P**lan individualization, with the question “Would the runner benefits from extension- or flexion-based exercises?”. The answers to these three questions are guidelines to create individualized exercise pathways based on our clinical experience, acknowledging that clinical studies are required to support our approach.

## The First Step of the PIMP Approach

**Figure 1:**
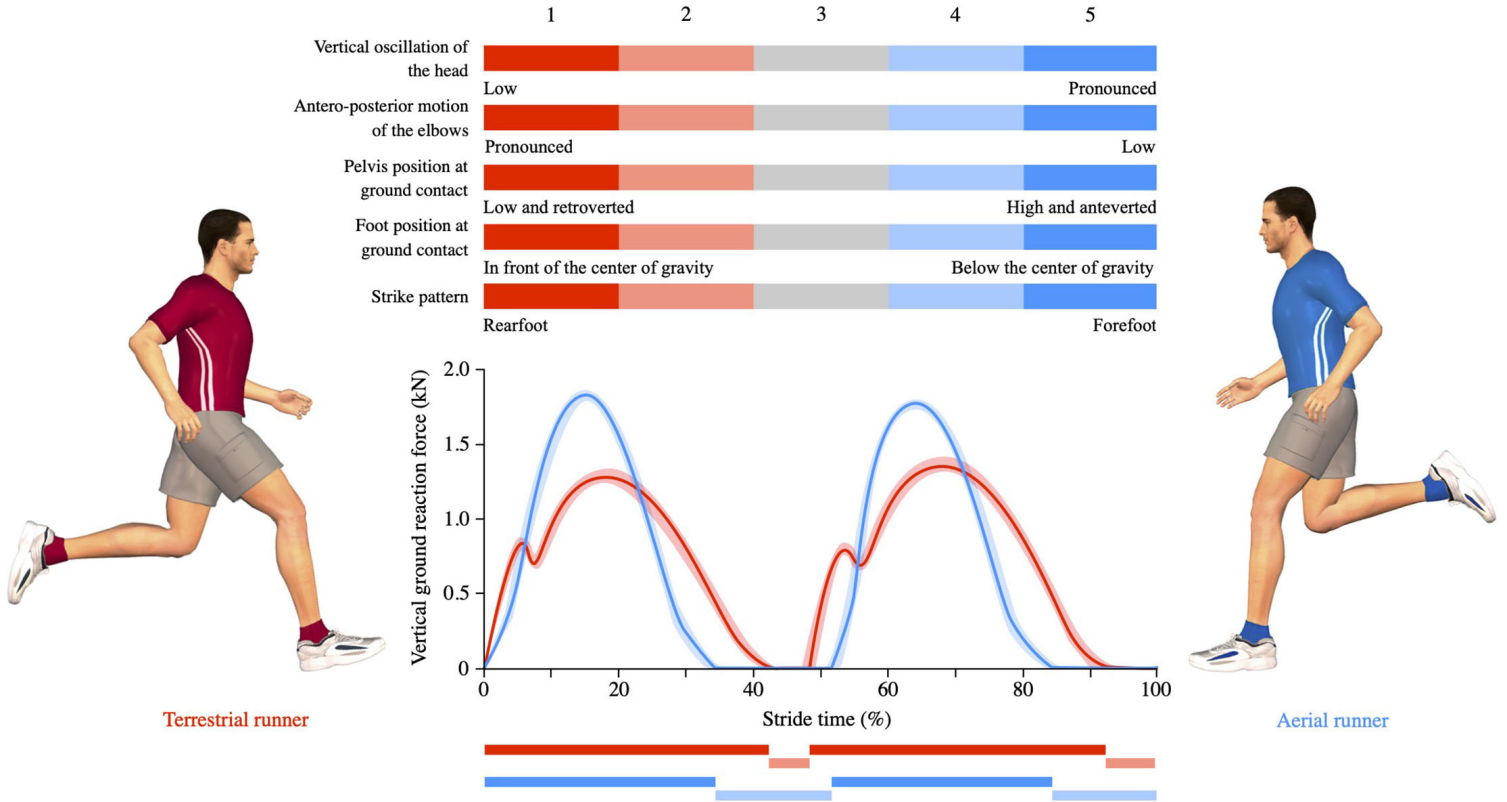
Schematic illustration of the Volodalen^®^ method used to evaluate the runner's running form and which attributes a global score ranging between 5 and 25 based on five criteria. Each of these five criteria is scored from 1 to 5. A global score smaller or equal to 15 indicates a terrestrial runner while a global score larger than 15 indicates an aerial runner. Illustration of posture and vertical ground reaction force during a running stride at 10 km/h in a typical flexed terrestrial runner (left picture and red curve) and a typical extended aerial runner (right picture and blue curve).

**Figure 2:**
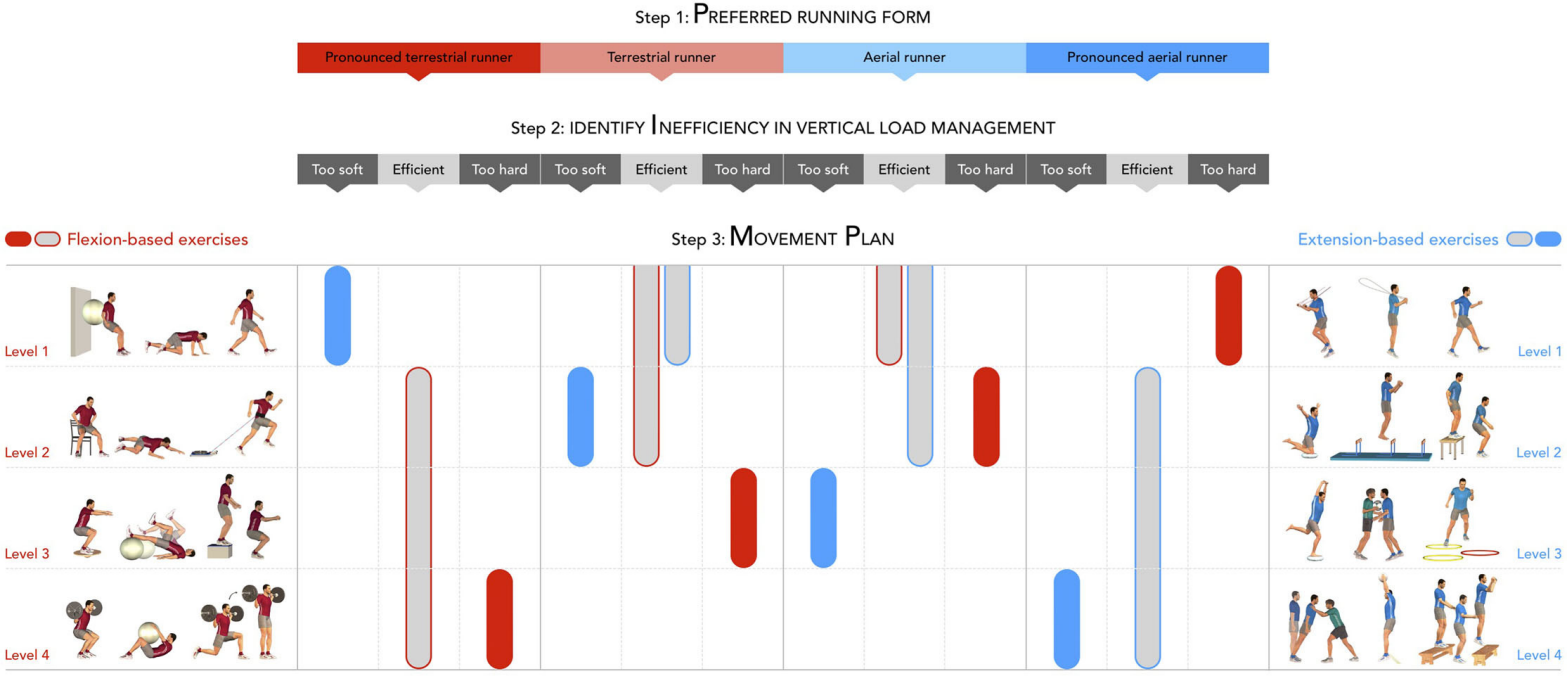
Schematic representation of the three steps (to be read vertically) of the PIMP approach. Exercises are ranked by their degree of flexion (left; red) or extension (right; blue) into four levels, with the fourth level showing the greatest degree of flexion or extension. This ranking allows individualizing the movement plan (step 3) based on the positioning of the runner along the terrestrial-aerial continuum (step 1) and the presence of a possible inefficiency in the vertical load management (step 2). Colored zones indicate if either flexion-based or extension-based are advised and too which degree (level 1–4). Gray areas indicate the proposed exercises for runners with efficient running forms (neither too soft nor too hard).

The first step in our approach is to determine the preferred running pattern. A wide range of running styles exists, with no unique style shown to be superior to another in terms of running endurance performance or injury risk (3, 6–8). Our research team has developed the Volodalen^®^ method which allows placing a runner's spontaneous running form along a continuum ranging from pronounced terrestrial to pronounced aerial. The Volodalen^®^ method comes from field observation and the principle of self-optimized movements. The method evaluates and scores five items to obtain a global V^®^score: vertical head oscillation, anterior-posterior motion of the elbows, pelvis position at ground contact, foot position at ground contact, and foot strike pattern (5) (Figure 1). These five items are subjectively scored by an expert from 1 to 5 and summed to obtain a quantitative V^®^score. In other words, a pronounced terrestrial running form shows limited vertical oscillation, pronounced arm movement, a pelvis position close to the ground, a foot strike position in front of the center of mass, and a rearfoot strike pattern. A pronounced aerial running pattern is characterized by the opposite. Based on the V^®^score, four categories can be determined: pronounced terrestrial (V^®^score range: 5–10), terrestrial (V^®^score range: 11–15), aerial (V^®^score range: 16–20), and pronounced aerial (V^®^score range: 21–25). The validity of the Volodalen^®^ method is supported by previous research (9, 10). Indeed, the visual observations of global running forms was shown to reflect quantifiable objective parameters (9). In addition, the method was shown to be a reliable tool to subjectively assess global running patterns, independently of the degree of expertise, whereas the subjective assessment of a single item of the V^®^score was rater-dependent (10). Alternatively, our research team showed that the duty factor (DF), the proportion of time spent in contact with the ground during a running stride (11), can be used as a laboratory-based and objective alternative to the subjective V^®^score (12). To summarize, a pronounced aerial running form is characterized by a spring-like running pattern with pronounced vertical oscillations and a more anterior (midfoot and forefoot) strike pattern than a terrestrial running form. In contrast, a pronounced terrestrial running form shows small vertical oscillations, as well as longer contact times, and a more rearfoot strike pattern than an aerial running form. Although the categorization and dichotomization of running forms always involve simplification, this practice is useful from a clinical perspective. For the PIMP approach, we propose clustering individuals into four categories (Figure 2): pronounced terrestrial—terrestrial—aerial—pronounced aerial. This categorization can be obtained using either the subjective V^®^score or the objective DF (Figure 1).

The importance of determining the preferred running pattern can be demonstrated by its relationship with metabolic cost. When comparing a group of aerial and terrestrial runners, both groups showed similar metabolic costs despite distinct running kinetics and kinematics (7, 13), in accordance with findings of similar metabolic costs for different running styles, as summarized elsewhere (6). This supports the idea that self-selected running forms are often the most economical (6), and that humans tend to self-optimize movement patterns to reduce metabolic cost. Another factor that shows the importance of preferred running pattern is its relationship with vertical load management, which leads to step 2.

## The Second Step of the PIMP Approach

The second step in our approach is to identify whether the runner or patient shows an inefficiency in the vertical load management. As running is a weight bearing activity, the way vertical load is handled is a key factor in RRI (2, 14). An efficient running stride comes from a compromise between compliance—the acceptance of joint deformation—and stiffness—the resistance against joint deformation. A terrestrial runner needs a certain degree of joint compliance to show a running pattern with less vertical oscillation and a smooth foot unroll. An efficient rearfoot strike needs sufficient ankle and knee range of motion to generate a smooth foot unroll during early stance (7, 14–16). On the contrary, an aerial runner needs a certain amount of leg stiffness to be able to perform a vertically oscillating stride. An efficient forefoot strike needs a sufficiently stiff ankle joint to be able to withstand the external ankle dorsiflexion moment during early stance (17, 18). However, both the aerial and terrestrial running pattern can become suboptimal in the vertical load management, which could lead to injury. Such inefficiencies can be categorized as either being “too soft”—an excessive compliance—or “too hard”—an excessive stiffness. Kinematic, kinetic, and spatiotemporal risk factors, as determined in a recent systematic review by Ceyssens et al. (2) were categorized according to the inefficiency identified by the PIMP approach (Table 1). Almost each risk factor could be interpreted as a sign of a too soft or too hard running pattern. For more details about the experimental conditions in which these data were collected, we refer the readers to the review and associated original articles.

**Table 1:** Kinematic, kinetic, and spatiotemporal risk factors as determined by a systematic review by Ceyssens et al. (2) and categorized according to the inefficiency identified by the PIMP approach.

**Inefficiency**	**Risk factor**	**Injured runner**	**Non-injured runner**	**Sex**
Too hard	↓ ankle eversion range of motion (°)	16.7 (2.5)	20.4 (3.7)	♀ / ♂
	↓ peak ankle eversion velocity (°/s)	326 (95)	479 (157)	♀ / ♂
	↑ knee joint stiffness (Nm/°)	6.89 (2.65)	6.72 (2.03)	♀ / ♂
	↑ vertical instantaneous loading rate (BW/s)	88.0 (13.9)	73.1 (15.9)	♀
		127 (40)	97 (31)	♂
	↑ vertical average loading rate (BW/s)	78.2 (11.1)	60.7 (12.8)	♀
	↑ vertical impact peak (BW)	1.72 (0.21)	1.51 (0.22)	♀
	↑ peak braking force (BW)	< -0.27	>-0.23	♀
	↓ step rate (over-striding) (steps/min)	<166	>178	♀ / ♂
	↓ ground contact time (s)	0.213 (0.040)	0.237 (0.026)	♂
Too soft	↑ peak hip adduction angle (°) (contralateral hip drop)	12.8 (2.8)	8.1 (4.5)	♀
	↑ internal knee abduction moment impulse (Nms)	9.2 (3.7)	4.7 (3.5)	♀ / ♂
	↑ peak external knee adduction moment (Nm/kg)	1.32 (1.08**–**1.56)	0.93 (0.78**–**1.08)	♀ / ♂
	↑ peak knee internal rotation angle	3.9 (3.7)	0.0 (4.6)	♀
	↑ peak ankle eversion velocity (°/s)	360 (271**–**449)	261 (212**–**310)	♀ / ♂
	↑ peak ankle eversion angle (°)	8.1 (3.0)	4.4 (4.2)	♀ / ♂
**Other**	↓ asymmetry in vertical impact peak (symmetry angle)	1.89 (1.88)	2.75 (2.48)	♀ / ♂
	↑ asymmetry in ground contact time (symmetry angle)	1.53 (1.04)	1.50 (2.06)	♀ / ♂

*Each risk factor is categorized as “too hard”, “too soft”, or other according to the inefficiency identified by the PIMP approach. Presented risk factors were found to have at least limited evidence of being related to running related injuries. Variables with very limited or no statistical relation with injury were not included. For each variable, mean (standard deviation) or mean (95% confidence interval) for the injured and non-injured runners were presented where possible. If not, cut-off values for high- and low-risk groups were given. In the last column, a male (♂) or female (♀) symbol was used to indicate whether evidence exists for male, female, or both*.

In running forms that are too hard, tissue vibrations or “noisy” strides linked to an excessive impact at ground contact are observed. For example, a runner that over-strides can be defined as having a too hard running pattern. Such a running pattern is characterized by a low step rate, an increased impact intensity, and large braking forces; all of which have been related to RRI (2). In contrast, inconsistencies in mobilities between transverse and coronal plane motion (especially at the feet, knees, and hips) and RRI conceptually underpin the too soft running form. In this case, the non-sagittal plane movements considerably contribute to impact attenuation. For instance, RRI such as the iliotibial band syndrome, can be associated with large ranges of non-sagittal motion, such as peak hip adduction (contralateral hip drop during stance) or knee internal rotation (19, 20). The excess of “softness” characteristics are more frequent in female runners, with a less clear association between non-sagittal plane biomechanics and RRI when considering both males and females (19).

These too soft and too hard characteristics can be observed in both aerial and terrestrial runners. The real challenge is to be able to observe these characteristics in a clinical setting. Most of these variables are only measurable in a laboratory setting, using equipment which most coaches, physiotherapists, or health clinicians do not have access too. However, it is possible to assess such motor inefficiency visually or by using cheaper technologies such as wearable sensors or video analyses. It must be noted that there is no clear threshold to define what constitutes too much or too little for any given biomechanical parameter. Besides, the values associated with the risk factors presented in Table 1 are speed, gender, method, and injury dependent. Hence, clinical judgment is essential in our proposed PIMP approach. Expertise in such clinical judgment can only be obtained through years of experience in assessing running gait parameters in uninjured and injured runners. The too soft or too hard concept should provide easy to interpret concepts for practitioners to develop such competence.

## The Third Step of the PIMP Approach

The third step in our approach consists in designing an individualized movement plan (Figure 2). **Extension-based strengthening exercises** promote short ground contact times, pushing the center of mass forward and upward, stiffness, and body alignment, i.e., shoulder-hip-knee-ankle, and activation of the posterior muscular chains. Such exercises correspond with how aerial runners manage the vertical load during running. Therefore, these exercises are suggested for aerial runners or any runners presenting a too soft running pattern. In contrast, **flexion-based strengthening exercises** promote long ground contact times, pushing the center of mass backward and downward, large ranges of motion, and activation of the anterior muscular chains. Such exercises correspond with how terrestrial runners manage the vertical load during running. Therefore, these exercises are recommended for terrestrial runners or any runners presenting a too hard running pattern. In other words, the preferred running form, as classified along the terrestrial-aerial continuum, determines the starting point of strengthening exercises (from flexion-based to extension-based). With these individualized exercises, we aim to allow the runners to PIMP their running form toward becoming less hard or less soft. The approach can be clarified with four examples, as presented in Table 2.

**Table 2:** Four examples of the application of the PIMP approach.

**Complaint or injury**	**Step 1** **Preferred running form**	**Step 2** **Identify inefficiency**	**Step 3** **Movement plan**
Lower back pain	Pronounced terrestrial	Too hard: overstriding	Level 4 flexion-based exercises *e.g.,: core stability in flexed position*
Proximal hamstring pain	Terrestrial	Too soft: increased transversal and frontal plane pelvic rotation	Level 2 extension-based exercises *e.g.,: step downs with external hip rotation*
Iliotibial band syndrome	Aerial	Too soft: knee valgus	Level 3 extension-based exercises *e.g.,: skipping drills*
Achilles tendinopathy	Pronounced aerial	Too hard: pronounced forefoot strike and increased flight times	Level 1 flexion-based exercises *e.g.,: quarter-squats*

Both a too soft or too hard running pattern have been linked independently with the same RRI (21). This highlights the importance of setting up a movement plan starting from the preferred running pattern and any inefficiency in the vertical load management, rather than only considering the type of injury. For instance, plantar fasciopathy has been related to both excessive pronation (too soft) and increased impact intensity (too hard) (21), warranting a different movement plan.

## Strengths and Limitations

The PIMP approach presented herein proposed to understand and analyze the running form from both a global and local point of view to enhance the ability of practitioners to individualize prescription, rehabilitation, and retraining programs, with the goal of minimizing the recurrence of running-related injuries. Such multiscale (global and local movements reading) approach could allow a better understanding of clinical, scientific, and social issues linked to recurrent running-related injuries. Indeed, physical and rehabilitation medicine is a real challenge in the 21st century (22) but the ease of use of the subjective approach presented herein makes it replicable in resource limited settings.

Nonetheless, the scientific validation of the effectiveness of the proposed approach is still needed. Indeed, the method is based on our clinical experience, biomechanical data, strength and conditioning knowledge, and empirical observations in both injured and uninjured runners. We acknowledge that further scientific justifications with appropriate clinical trials and mechanistic research are required to substantiate the approach and therefore constitute the main limitation of the method in its current form. Moreover, determining the preferred running pattern of injured runners might be a difficult task because of possible gait modifications due to pain. In that case, indirect information could be obtained, e.g., by looking at the wear patterns of the shoes, by assessing the antero-posterior position of the quiet standing center of pressure (23), or by asking how runners perceive their running form.

## Perspectives

In addition to strengthening exercises, gait retraining, e.g., stride frequency or foot strike pattern manipulations, can be an important part of the rehabilitation program (24). The PIMP approach can also be used to guide gait retraining strategies and recommendations but elaborating on this PIMP application is beyond the scope of the current opinion and warrants a separate discussion. We believe the presented approach provides a general framework for practitioners to evaluate preferred running forms, identify inefficiency in vertical load management, and design an individualized movement plan.

## Data Availability Statement

The original contributions presented in the study are included in the article/supplementary material, further inquiries can be directed to the corresponding author/s.

## Author Contributions

CG was responsible for the conceptualization of the approach. All authors contributed to the article and approved the submitted version.

## Funding

This work was supported by the Volodalen Company, the Université of Franche Comté and Tomsk Polytechnic University Development Program.

## Conflict of Interest

CG is the originator of the Volodalen^®^ method. CG, BB, and TL are employed by Volodalen. AT was employed by Volodalen at the time of writing the manuscript. The remaining authors declare that the research was conducted in the absence of any commercial or financial relationships that could be construed as a potential conflict of interest.

## Publisher's Note

All claims expressed in this article are solely those of the authors and do not necessarily represent those of their affiliated organizations, or those of the publisher, the editors and the reviewers. Any product that may be evaluated in this article, or claim that may be made by its manufacturer, is not guaranteed or endorsed by the publisher.
